# The Role of Co-Stimulatory Molecules in Chagas Disease

**DOI:** 10.3390/cells7110200

**Published:** 2018-11-07

**Authors:** Bruna F. Pinto, Nayara I. Medeiros, Tereza C. M. Fontes-Cal, Isabela M. Naziazeno, Rodrigo Correa-Oliveira, Walderez O. Dutra, Juliana A. S. Gomes

**Affiliations:** 1 Institute of Biological Sciences, Department of Morphology, Federal University of Minas Gerais, Belo Horizonte, Minas Gerais 31270-901, Brazil; bruna_fernandes_6@hotmail.com (B.F.P.); t.t.cal@hotmail.com (T.C.M.F.-C.); isabelamarques_s21995@hotmail.com (I.M.N.); wdutra@icb.ufmg.br (W.O.D.); 2 René Rachou Institute, Oswaldo Cruz Foundation, Belo Horizonte, Minas Gerais 30.190-009, Brazil; naymedeiros@yahoo.com (N.I.M.); correa@minas.fiocruz.br (R.C.-O.); 3 National Institute of Science and Technology in Tropical Diseases, INCT-DT, Belo Horizonte, Minas Gerais 31270-901, Brazil

**Keywords:** Chagas disease, monocytes, lymphocytes, co-stimulatory molecules

## Abstract

Chagas disease, caused by *Trypanosoma cruzi*, is a potentially life-threatening tropical disease endemic to Latin American countries that affects approximately 8 million people. In the chronic phase of the disease, individuals are classified as belonging to the indeterminate clinical form or to the cardiac and/or digestive forms when clinical symptoms are apparent. The relationship between monocytes and lymphocytes may be an important point to help clarify the complexity that surrounds the clinical symptoms of the chronic phase of Chagas disease. The co-stimulatory signals are essential to determining the magnitude of T cell response to the antigen. The signals are known to determine the regulation of subsequent adaptive immune response. However, little is known about the expression and function of these molecules in Chagas disease. Therefore, this review aims to discuss the possible role of main pathways of co-stimulatory molecule-receptor interactions in this pathology that could be crucial to understand the disease dynamics.

## 1. Introduction: A Brief Overview of Chagas Disease

Although Chagas disease, which is caused by the protozoan *Trypanosoma cruzi* (*T. cruzi*), was discovered in 1909 by Carlos Ribeiro Justiniano das Chagas, it remains a serious public health problem in many countries. Currently, there are 8 million people infected by the parasite all around the world, mainly in 21 countries in Latin America where the disease is endemic [1,2,3]. It is estimated that 10,000 deaths of *T. cruzi*-infected people occur per year, and more than 25 million individuals are at the risk of infection [4].

Since the early 1990s, the most effective measures to control Chagas disease in Latin America have been through vector control programs and compulsory blood bank testing [2,3,5]. The Southern Cone Initiative (Iniciativa de Salud del Cono Sur, INCOSUR) was launched in 1991 to eliminate the main vector, *Triatoma infestans*, and transmission by blood transfusion in Argentina, Bolivia, Brazil, Chile, Paraguay, and Uruguay [6]. This initiative led to a reduction in *T. cruzi* transmission, and its interruption was certified in Uruguay (1997), Chile (1999), Argentina (2001), Brazil (2000) and Paraguay (2002) [2,7]. However, despite these efforts to combat the transmission of the disease in still endemic regions in Latin America, Chagas disease has become a significant epidemiological, economic and social problem at the global level [8] due to the migration of infected individuals from endemic regions to non-endemic countries in North America, Europe, Asia, and Oceania [4,9,10,11].

In addition to vector transmission, the parasite can be transmitted to humans through blood transfusion, organ transplantation, laboratory accidents, congenitally, and through ingestion of food contaminated with trypomastigote forms of *T. cruzi* [11,12,13]. These forms of transmission are responsible for the introduction and maintenance of Chagas disease in non-endemic regions and contribute to the persistence and re-emergence of the disease in endemic countries [14,15]. Oral infection currently represents the most prevalent transmission route in Brazil [11,16]. The Ministry of Health recorded 1252 cases of acute Chagas disease between 2007 and 2014 in Brazil, approximately 70% of which were due to oral transmission [17].

Chagas disease is characterized by two distinct phases: the acute phase, which may last between one and two months, and the chronic phase. In the acute phase, most cases are asymptomatic, for about 50% of infected individuals, or oligo-symptomatic, when some clinical manifestations are evident, such as fever, generalized adenopathy, edema, hepatosplenomegaly, or myocarditis [18]. However, in other cases, classic symptoms of the disease may be apparent, such as signs of portal entry, defined by edema at the infection site or Romaña signal, characterized by unilateral palpebral edema in the ocular conjunctiva [18,19]. This phase is also characterized by an increase in parasitemia due to intense parasite multiplication inside the host cells [10,20] and death due to severe complications [19]. After the acute phase, there is a decrease in parasitemia due to the host immune response and the infection progresses to the chronic phase [10,19]. About 60% of infected individuals develop the indeterminate clinical form (IND), characterized by positive serological tests and the absence of clinical manifestations [10,18,20,21,22,23]. Patients with the IND clinical form may not develop severe clinical manifestations and remain asymptomatic throughout their lives [12]. However, over time, asymptomatic individuals may develop symptoms and evolve to the symptomatic clinical form [24]. Approximately 30% of infected individuals develop the cardiac clinical form (CARD), characterized by myocarditis, destruction of cardiac fibers in the inflammatory focus, fibrosis, cardiomegaly, and congestive heart failure, which can cause the sudden death of the patient [20,24,25,26]. Heart failure caused by chronic Chagas cardiomyopathy has the worst prognosis and a survival rate of less than 50% when compared to other heart diseases [23,27]. The digestive clinical form of Chagas disease (DIG) affects approximately 10% of infected individuals [10]. This form is characterized by gastrointestinal disturbances that may lead to megacolon and/or megaesophagus formation [28]. The cardiodigestive clinical form (CDG), also known as the mixed form, is characterized by clinical symptoms compatible with CARD and DIG forms simultaneously [10,29,30].

Patients with the acute phase of the disease present high parasitemia and the trypomastigotes can be detected through blood microscopy. In this phase, the evolution or regression of the parasite load may be monitored using the Polymerase Chain Reaction, which offers both a qualitative and a quantitative assessment of the *T. cruzi* burden. The transition from the acute to the chronic phase is accompanied by a marked decrease in parasitemia, as a result of the host’s immune response. In this phase, diagnosis focuses on the detection of serum antibodies to the parasite, for which there are three serologic tests: indirect hemagglutination; indirect immunofluourescence; and enzyme-linked immunosorbent assay [31].

The mechanisms involved in the development of severe forms of Chagas disease are not yet well understood. However, the involvement of the host immune responses mediated by monocytes and lymphocytes has been shown to be crucial in determining the disease pathogenesis [32,33,34,35]. Monocytes are innate immune cells that recognize pathogen-associated molecular patterns (PAMPs) from the parasite through Toll-Like receptors such as TLR-2, 4, and 9 [36,37,38]. These cells activate the subsequent adaptive immune response by processing and presenting *T. cruzi* antigens by major histocompatibility complex II (MHC-II) to CD4^+^ T cells and major histocompatibility complex I (MHC-I) to CD8^+^ T cells [39]. However, to effectively activate T lymphocytes, the interaction of accessory co-stimulatory molecules that will provide a signal to activate the adaptive immune response is essential.

CD8^+^ T lymphocytes play a crucial role during the acute phase of the pathology. These cells produce IFN-γ to activate effector mechanisms in macrophages to destroy amastigotes forms of *T. cruzi*, even as display cytotoxic activity to destroy cells infected with intracellular amastigotes [36]. On the other hand, the activation of T CD4^+^ lymphocytes may result in a functional differentiation into Th1, Th2, Th17, or Treg effector cells that differ in terms of their cytokine secretion and trigger distinct immune responses in Chagas disease [14].

Many studies have shown that patients with Chagas cardiomyopathy develop an exacerbated pro-inflammatory cytokine environment, such as IFN-γ e TNF-α, which directs the Th1 lymphocyte-mediate response. In addition, these patients also showed a low frequency of regulatory cells and anti-inflammatory cytokines that cannot control the exacerbated response that leads to a loss of regulation of immune response and contributes to pathology maintenance [27,32,34,40,41,42]. Conversely, patients with the asymptomatic clinical form, despite having an important Th1 inflammatory response to control parasite replication, largely produce anti-inflammatory cytokines, such as IL-10, showing a balance between inflammatory and modulating cytokines production that controls tissue damage and leads to a more modulated T lymphocyte-mediated response [11,24,32,34,43,44,45,46].

Although many studies have demonstrated the importance of cellular immune response in the clinical dichotomy of Chagas disease [32,35,39,47,48,49], little is known about the role of co-stimulatory molecules in pathology development. Therefore, this review focuses on describing the co-stimulatory molecules and their possible role in Chagas disease.

## 2. Co-Stimulatory Molecules and Their Role in Chagas Disease

The immune response is essential to protecting the organism against a plethora of infections, inducing multiple humoral and cellular mechanisms of the innate and adaptive immune responses. CD4^+^ T cell activation requires two signals: the first is provided by the interaction of MHC-II with the T cell receptor (TCR); and the second is delivered to T cells by co-stimulatory cell surface molecules expressed on antigen-presenting cells (APCs) [50].

The B7-1/B7-2–CD28/CTLA-4 pathway is the best-characterized co-stimulatory interaction, being crucial to T lymphocytes activation [51,52]. The B7 family is composed of members of the immunoglobulin family that display V and C-like domains [50]. The most well-known members of this family are B7-1 (also known as CD80) and B7-2 (also known as CD86), which interact with two members of the CD28 family, the CD28 and cytotoxic T-lymphocyte–associated antigen 4, CTLA-4 (also known as CD152) receptors [52,53]. However, other members of the B7-CD28 superfamily have been studied, such as programmed cell death protein 1 (PD-1), PD-L1 (also known as B7-H1), PD-L2 (also known as B7-DC and CD273), inducible T-cell co-stimulator (ICOS) (also known as H4 and AILIM), and ICOSL (also known as KIAA0653, B7h, GL50, B7RP-1, LICOS, and B7-H2) [50]. In addition, many co-stimulatory pathways have been identified, including CD40L (also known as CD154)/CD40, CD2 (also known as LFA-2)/CD58 (also known as LFA-3), LFA-1 (also known as CD18)/ICAM-1 (also known as CD54), CD95 (also known as Fas)/CD95L (also known as FasL), and the Tumor necrosis factor family (TNF)/TNF receptor family (TNFR) [51,52].

Several studies have evaluated the role of co-stimulatory molecules in the immune response in different pathological contexts [54,55,56,57]. Thus, co-stimulatory molecules play a pivotal role in determining the outcome of T cells, directing the activation or inhibition of lymphocytes and the fate of the immune response. Here, we will describe the main co-stimulation receptors and their possible role in Chagas disease.

### 2.1. PD-L1, PD-L2, and PD-1 Receptors

PD-1 belongs to the CD28 family and interacts only with two B7 homologues, the PD-L1 and PD-L2 receptors [50]. This receptor is expressed by activated, but not resting, T cells, B cells, and myeloid cells [58,59], while its ligands are expressed in APC, with PD-L1 expressed mainly on B cells, dendritic cells (DCs), and monocytes, and PD-L2 are expressed only on DCs [50].

It has been proposed that the interaction between PD-1 and PD-L1 or PD-L2 inhibits the activation of T cells, suppressing T-cell function and downregulating the immune response [56,58]. In addition, PD-1 is overly expressed on exhausted T cells and the homeostasis of the immune response is returned upon the PD-1 or PD-L1 blockade [52]. Some studies have shown that C57BL/6 PD-1^(−/−)^ mice developed lupus-like proliferative arthritis and glomerulonephritis, while BALB/c PD-1^(−/−)^ developed a fatal dilated cardiomyopathy with thrombosis [54,55]. Other studies have shown that anti-PD-1 antibodies could be efficient therapies in cancer treatment [52,57]. Taken together, these results indicate that the PD-1 pathway is an important negative regulator of the immune system and that the absence of this receptor can lead to autoimmunity.

Nevertheless, little is known about the PD-1 function in Chagas disease. Stempin et al. (2017) reported an increase in the expression of PD-1 spleen CD4^+^ T cells from mice infected with the Tulahuen strain of *T. cruzi* at 21 days after infection. However, PD-1 expression decreased significantly at 42 days after infection [60]. In addition, another study evaluated the expression of PD-1L on DCs from C57BL/6 mice infected with four different *T. cruzi* strains and observed that all strains were able to induce the expression of PD-L1 after 18 h of DCs infection. Conversely, only the 2369 strain induced an increase in IL-10 anti-inflammatory molecules and PD-L1 expression, but not of TNF-α, MHC-II, or CD40 [61]. The frequency of polymorphism of the PDCD1 gene that encodes the PD-1 receptor, the PD-1.3G/A, was evaluated and correlated with patients with cardiac (*n* = 90), digestive (*n* = 67), cardiodigestive (*n* = 39), and indeterminate (*n* = 81) clinical forms of chronic Chagas disease [62]. However, no statistical difference in the PD-1.3G/A polymorphism was observed between the different clinical forms of Chagas disease and healthy controls. However, another study observed a higher frequency of CD8^+^ PD-1^+^ T cells in patients with chronic Chagas disease when compared to healthy donors. In addition, a higher frequency of CD8^+^ T cells expressing PD-1 in symptomatic versus asymptomatic patients was detected [63].

Borges et al. (2012) observed a higher expression of the inhibitory PD-1 molecule in CD4^+^ CD25^+^ T cells in the spleen of mice infected with the intermediate and higher inoculum of the Colombian strain (3000 and 30,000 parasites, respectively). However, in the intermediate inoculum, the PD-1 receptor was associated with the control of inflammation and in the higher inoculum, the presence of this inhibitory molecule on the spleen was not sufficient to control inflammation and avoid mice mortality [64]. Another study showed that PD-1 and its ligands were highly expressed in lymphocytes found in the hearts of mice infected with the Y strain of *T. cruzi*, and the blockade or deletion of this receptor increased the proliferative response by lymphocytes, acute myocarditis, and mice mortality [65].

The expression of PD-1, PD-L1, and PD-L2 was evaluated in peritoneal macrophages of mice infected with the Tulahuen strain, and a different role of these co-stimulatory molecules was seen. An increase in nitric oxide (NO) production was observed in cells from infected mice treated with anti-PD-1 or anti-PD-L. Even so, anti-PD-L2 blocking antibody treatment reduces iNOS expression and NO production by infected macrophage [66]. Thus, the PD-2L receptor might play a protective role in the immune response against *T. cruzi*.

### 2.2. ICOSL and ICOS Receptors

ICOS is a co-stimulatory receptor present on the T cell surface [52,67,68] that interacts with ICOSL, which is constitutively expressed on B cells, macrophages, and DCs [68,69]. It has been proposed that ICOSL binds strongly to ICOS at 37 °C, but, can bind weakly to CD28 e CTLA-4 at a lower non-physiological temperature (25 °C) [70].

In contrast to the CD28 receptor, ICOS is not constitutively expressed, showing a low expression on naive T cells. However, it is rapidly induced on T cells after TCR engagement [50,68]. Initially, it was known that ICOS was restricted to T cell activation and T cell-dependent B cell responses by interacting with its ligand, ICOSL. However, ICOSL can also modulate the immune response, promoting the T cell stimulatory and inhibitory pathways through its interaction with CD28 and CTLA-4, respectively [69,71].

It has been proposed that the binding of ICOS with ICOSL is of great importance to T cell activation and differentiation. Dong et al. (2011) investigated the role of the ICOS receptor in T-cell activation in ICOS-deficient mice. Purified naive CD4^+^ T cells from ICOS knockout mice incubated with wild-type APCs displayed a reduced expression of IL-2. It was also shown that differentiated Th1 and Th2 CD4^+^ T lymphocytes were able to express IFN-γ and IL-10, but they failed to produce IL-2 and IL-4, the latter being an essential cytokine to Th2 differentiation [72]. Thus, ICOS is essential to IL-2 production by CD4^+^ T lymphocytes being crucial for T cell activation and proliferation.

The role of the ICOS/ICOSL pathway in *T. cruzi* infection is not well understood, but ICOS plays a crucial role in T cell responses in other infectious diseases caused by protozoans [73,74,75]. A previous study has shown that CD28^−/−^ BALB/c mice infected with *Toxoplasma gondii* (*T. gondii*) developed a T-cell response that was sufficient to provide resistance to the infection [73], suggesting that the ICOS receptor is an excellent candidate for inducing a CD28-independent activation of T cells. Villegas et al. (2002) observed that in vivo blocking of the ICOS co-stimulatory pathway in CD28^−/−^ mice infected with *T. gondii* leads to a decrease in IFN-γ serum levels, and an increase in parasite burden and mortality when compared to the control group [74]. Additional studies have shown that ICOS^−/−^ mice infected with *Leishmania mexicana* showed a reduction in IL-4 e IFN-γ production when compared to wild-type mice [75]. These studies suggest that ICOS may be involved in Th1 and Th2 regulation, and is crucial in T helper cell differentiation.

### 2.3. Tumor Necrosis Factor Family

So far, 29 members of the TNF receptor superfamily have been identified in humans and, according to their cytoplasmic sequences and signaling pathway, they can be divided into three major groups [76,77]. The first group includes receptors with a death domain (DD) in the cytoplasmic tail, such as Fas (CD95), TNF-R1, DR3, TRAIL-R1, TRAIL-R2, and DR6 receptors [76]. The interactions of these receptors with their ligands lead to recruitment of adaptor molecules such as Fas-associated DD (FADD) and TNFR-associated DD (TRADD), which activate the caspase cascade and lead to cell death by apoptosis [76,77].

The second group includes TNF-R2, CD40, CD30, CD27, LTβR, Ox40, 4-1BB, BAFF-R, BCMA, TACI, RANK, p75NGFR, HVEM, TNFRSF18, TROY, EDAR, XEDAR, RELT, and Fn14 receptors, which contain one or more TNF receptor associated factor (TRAF)-interacting motifs (TIMs) in their cytoplasmatic tails. These receptors activate TIM through the interaction with their ligands, which recruit TRAF family members and lead to the activation of different signal transduction pathways, such as nuclear factor κB (NF-κB) and phosphoinositide 3-kinase (PI3K), the well-known signaling pathways. The recruitment of TRAF is associated with cellular activation, differentiation, survival, and death [76,78,79].

The last group includes TRAIL-R3, TRAIL-R4, decoy-R3, and osteoprotegerin (OPG) receptors, also known as “decoy receptors”. Differently from the other groups of the TNF receptor family, this one does not contain functional intracellular signaling domains or motifs, meaning that these receptors cannot trigger intracellular signaling pathways and can only compete with the binding sites of the other groups. Therefore, the effective function of these receptors is to block the activation of signal transduction pathways of the other TNF receptor groups [76].

The TNF superfamily is constituted not only by receptors, but also by binders that form the TNF ligand family (TNFSF) [80]. This family comprises 18 genes encoding 19 type II transmembrane proteins defined by structural homology in their ectodomain. This domain is composed by trimers that form a highly efficient receptor clustering responsible for the interaction with their specific receptors [80,81]. Although all ligands are synthesized as transmembrane proteins, some are cleaved by proteases and released as soluble forms [81]. Among the TNF ligands, we can cite Lymphotoxin α and Lymphotoxin β, being the first one secreted as a homotrimer or which can be anchored to the to the activated T lymphocytes membrane as a heterotrimer with Lymphotoxin β, a type II transmembrane protein. These proteins are related to the embryonic development of peripheral lymph organs and the formation of germinal centers in adults during immune responses [82].

TNFR/TNF interactions play an important role in the immune responses involved in the activation of APCs, as also provide co-stimulatory signals to T cells [83,84]. Many studies have shown the role of these receptors as co-stimulatory molecules in different pathological contexts [77,79,84,85,86,87,88]. However, only a few studies have showed the role of these molecules in Chagas disease. In *T. cruzi* infection, C57BL/6 mice infected with the parasite showed high levels of CD40 co-stimulatory molecule by DCs and macrophages. In addition, CD3^+^ T cells from these infected mice also demonstrated high IL-2 and IFN-γ production [89]. Another study showed a higher CD40 expression in HL-1 murine cardiomyocytes infected with *T. cruzi*, and the interaction of this receptor with its CD40L ligand on lymphocytes infiltrating in the heart can induce IL-6 production and contribute to tissue inflammation [90]. Transfection of 3H3 fibroblasts-CD40L infected with *T. cruzi* leads to the control of parasitemia through a NO-mediated IL-12-dependent pathway [91]. Another study showed that blockage of the IFN-γ or CD40 receptor on human monocytes infected with Y strain of *T. cruzi* causes 50% of the inhibition of the IL-12 expression, suggesting that most of the production of this pro-inflammatory cytokine is due to IFN-γ and CD40-CD40L interactions [92].

These results indicate that CD40 receptor engagement is crucial to controlling parasite replication by the production of the IL-2, IL-6, and IL-12 pro-inflammatory cytokines. However, CD40 receptor interaction can also modulate the immune response by IL-10 anti-inflammatory cytokine production and activation of Tregs, as seen in Leishmania infection [93].

In other infectious diseases caused by protozoans, such as Leishmania, the CD40 receptor is involved in the activation of macrophages and the production of IL-12 by DCs. CD40^−/−^ mice infected with *Leishmania major* (*L. major*) showed a swift development of the disease when compared to the susceptible control group, triggering a Th2 lymphocyte response characterized by higher IL-4 levels [94]. It has been shown that IFN-γ stimulates infected macrophages to produce high NO, which is crucial to potentiate the lysis of the protozoan [1]. However, macrophages infected with *L. major* and treated with exogenous IFN-γ are unable to effectively clear the parasites [95]. Kamanaka et al. (1996) showed that the addition of anti-CD40 antibody in conjunction with IFN-γ treatment is a crucial signal to macrophage activation, contributing to a lower parasite burden [94]. Moreover, CD40L^−/−^ mice also displayed a defective T cell response, evident when peritoneal macrophages from CD40 knockout mice treated with soluble CD40L and IFN-γ demonstrated an increase in IL-12 production [96]. These results suggested that CD40 is pivotal for the development of an inflammatory response that contributes to parasite elimination. Nevertheless, previous studies have shown that different levels of CD40 expression on DCs can be associated with the development of different T cell subsets. Higher levels of CD40 expression trigger the development of effector T cells that contribute to parasite clearance, whereas lower CD40 expression levels trigger regulatory T cell (Treg) development. In addition, the binding of CD40 with its ligand can favor the production of IL-12 or IL-10 cytokines [93,95].

Apoptosis is a mechanism of programmed cell death that can occur in physiological and pathological contexts [97]. In myocardial diseases, due to the limited capacity of cardiomyocyte regeneration, cardiac cell apoptosis can contribute to ventricular dysfunction and heart failure [98,99]. In Chagas disease, it has been demonstrated that cardiomyocyte samples from chronic Chagas cardiomyopathy patients undergo apoptosis when compared to Chagas patients without heart failure. Apoptosis contributes to myocardial cell loss and the development of severe heart damage [100]. Furthermore, apoptosis has also been observed in the inflammatory cells from patients with heart failure, and this cascade of cellular apoptosis could be mediated by the TNF receptor superfamily, such as CD95/CD95L interaction [99]. The CD95/CD95L engagement promotes apoptosis of CD4^+^ T lymphocytes co-cultivated with macrophages infected with *T. cruzi*, leading to the exacerbated parasite growth in the acute infection. However, binding CD95 to its ligand is also essential to immune response control [97]. Contrarily, IL-10 e IL-4 production was seen to be enhanced, but IL-2 e IFN-γ pro-inflammatory cytokines in supernatants from T cell cultures treated with anti-CD95L from mice infected with *T. cruzi* [101] were not seen to increase. It was also observed an increased secretion of Th2 cytokines IL-10 and IL-4 by CD4^+^ T cells in CD95L-deficient BALB gld/gld mice infected with *T. cruzi* [102]. Other studies showed high CD95L expression in CD4⁺ CD95⁺ T lymphocytes from IND patients when compared to the NI group. However, after *T. cruzi* stimulation, a decrease in CD95L expression in CD4^+^ CD95^+^ T cells only in IND patients [103] was seen and higher levels of CD95L receptor in patients with Chagas disease were also observed, regardless of the clinical form [99]. All these results suggest that, in *T. cruzi* acute infection, CD95/CD95L interaction leads to the apoptosis of immune cells, such as CD4^+^ T lymphocytes, which cannot control parasite multiplication and, therefore, contribute to the persistence of the infection. We think blocking these receptors during Chagas disease development could be essential to down-modulate the immune response and control exacerbated inflammation.

### 2.4. CD2 and CD58 Receptors

CD2, an adhesion molecule, belongs to the CD2 subfamily and, along with the CD150 family, falls in the immunoglobulin superfamily (IgSF) [104,105]. This receptor is not only expressed in red blood cells (RBC), but is also constitutively expressed on T lymphocytes [106,107,108] and interacts with CD58 (a glycoprotein presents mainly in APCs). Many studies have shown the role of CD2 in T-cell activation, with this receptor being a positive regulator of T-cell signaling [106,108]. The activation of T cells by CD2 signaling involves this receptor directly interacting with the TCR-CD3 complex and, in this co-engagement, CD2 is considered as a co-stimulatory molecule [104,107]. However, it has been demonstrated that the CD2 co-stimulatory molecule can activate T cells in the absence of TCR, being an alternate pathway to the activation of lymphocytes [106,109].

Further studies have shown that T cells from CD28-deficient mice, a co-stimulatory molecule essential to the second signal to T-cell activation [110] were still capable of inducing an immune response [111]. Moreover, while CD2 or CD28-deficient mice showed mild expression defects, when CD2/CD28 double-deficient mice were evaluated, they showed major defects in lymphocyte activation and proliferation. These results suggest that both receptors—CD2 and CD28—have similar functions in the activation of T cells and that together they are capable of regulating initial steps in T cell activation [107].

Recent studies have evaluated these receptors in different pathological contexts [112,113,114,115,116,117,118]. Co-cultures with blood forms of the Tulahuen strain of *T. cruzi* and human peripheral blood mononuclear cells stimulated with anti-CD2 monoclonal antibodies have shown a reduction in the proliferative capacity of lymphocytes and a lower expression of IL-2R when compared to cultures without the parasite [119]. A lower expression of CD58 was also observed in lymphocytes in the heart of patients with cardiac Chagas disease [120]. All these findings suggest that the immune response is down-modulated in the presence of unicellular parasites by reduced expression of CD2 on T cells. This, in turn, decreases lymphocytes activation and contributes to the persistence of infection.

### 2.5. LFA-1 and ICAM-1 Receptors

LFA-I is the most abundant and widespread integrin expressed in all human T cells [109,121], playing a pivotal role in T lymphocytes function and mediating cell adhesion, the migration of T cells from the lymph nodes to the inflammatory sites, and antigen presentation [121,122]. In addition to interacting with members of the immunoglobulin superfamily present on the endothelium known as an intercellular adhesion molecule (ICAM)-1, ICAM-2, vascular cell adhesion molecule (VCAM)-1, and mucosal addressin cell adhesion molecule (MAdCAM)-1 [121], LFA-I also interacts with ICAM-1 present on the antigen-presenting cell surface [123]. The expression of ICAM-1 on DCs and its interaction with LFA-1 are crucial for effective T cell activation [123]. It was observed that LFA-1/ICAM-1 engagement can induce Th17 cells in experimental autoimmune encephalomyelitis and also contributes to iTreg differentiation [124,125,126]. In contrast, the interaction of the LFA-1 with ICAM-1 T can promote Th1 polarization through the increase in IL-2 and IFN-γ pro-inflammatory cytokines [126]. These results suggest that the engagement of these receptors could modulate the immune response and play an important role in inflammation and homeostasis.

A higher expression of LFA-1 was observed in the inflammatory infiltrate on the myocardium of *T. cruzi*-infected mice [127,128]. A higher expression of LFA-1 in lymphocytes in the heart of Chagas patients with cardiac disease was also observed [120]. Conversely, another study showed an increased LFA-1 expression on invading cells from cardiac tissue of *T. cruzi*-infected animals with the Colombian strain at 28 days after infection. Moreover, the inflammatory infiltrate was constituted mainly by CD8^+^ T lymphocytes and less frequently by CD4^+^ T cells [129]. Ferreira et al. (2017) observed that LFA-1 played a pivotal role in CD8^+^ T cell migration and could be related to the cytotoxicity of CD8^+^ T cells [130].

An increase in ICAM-1 expression has been demonstrated not only in the inflammatory infiltrate leukocytes, but also in cardiomyocytes in acute infection with different strains of *T. cruzi*. Furthermore, an increase in this receptor expression was paralleled by the production of pro-inflammatory cytokines [131], as an increase in the ICAM-1 expression on circulating mononuclear cells during the early acute phase of *T. cruzi* infection was also observed [129]. Together, all these findings emphasize that the interaction of LFA-1 and ICAM-1 is essential for inflammation and could be important in activating and moving T cells to inflammatory infiltrate during acute *T. cruzi* infection. It is also possible that this engagement could be a strategy employed by *T. cruzi* to attract cells towards the inflammatory foci.

### 2.6. Role of CD28, CTLA-4, CD80, and CD86 Co-Stimulatory Molecules in the Pathogenesis of Chagas Disease

CD4^+^ T lymphocytes are important cells in the adaptive immune response whose activation requires at least two distinct signals: signals mediated by MHC-II e TCR receptors; and a second signal by the co-stimulatory molecules [51,132]. It has been demonstrated that the interaction of TCR with peptide antigen bound to MHC-II is not capable of inducing T cell activation and the engagement of co-stimulatory molecules is crucial for effective lymphocyte activation e proliferation [110,133,134].

B7-1, B7-2, CD28, and CTLA-4 receptors are the most thoroughly studied co-stimulatory molecules essential for the activation/inhibition of adaptive immunity [52]. B7-1 and B7-2 are present on the cell surface of professional antigen-presenting cells (APC), such as DCs, B lymphocytes, monocytes, and macrophages, and interact with CD28 and CTLA-4 receptors present on the CD4^+^ T lymphocyte cell surface [135]. All these co-stimulatory molecules are differently expressed with their distinct functions. While the CD28 receptor is constitutively expressed on T cells and shows low avidity for B7 molecules, CTLA-4 is rapidly upregulated only after T cell activation [50,136,137]. The interaction of CD80 and CD86 with CD28 triggers the activation of T cells, promoting lymphocyte proliferation, IL-2 production, and consequently an enhanced immune response, whereas binding to CTLA-4 inhibits the activation of lymphocytes, which down-regulates immunity [39,137,138,139].

The B7-2 co-stimulatory molecule is constitutively expressed at low levels on APCs, whereas B7-1 is upregulated later only after activation [50,139,140]. These molecules have shown a large overlap of functions [50]. Differences in B7-1 e B7-2 functions may be related to the time they are expressed in the cells. B7-2 is expressed early and can mediate the initial immune response by interacting with the CD28 receptor and B7-1 might play an important function in late inflammation.

The CTLA-4-deficient mice showed a higher lymphocyte infiltrate into various organs, evidencing a high proliferative capacity of these cells with early lethality [141]. CD28-deficient mice decrease the activation of T-helper lymphocytes, but delayed-type hypersensitivity and cytotoxic T-cell activity could still be induced [142,143]. June et al. (1990) observed that the CD28 co-stimulatory molecule can trigger T-cell activation or anergy [144]. Moreover, blocking B7/CD28 interactions results in antigen-specific unresponsiveness [145].

Many studies have demonstrated the functional role of CD80, CD86, CTLA-4, and CD28 receptors in different pathological settings [137,139,146,147,148,149], yet little is known about the role of these co-stimulatory molecules in Chagas disease patients. A few studies have shown that distinct strains of *Trypanosoma cruzi* could trigger different mechanisms of activation of the immune response mediated by monocytes and lymphocytes. Magalhães et al. (2015) observed a higher CD80 and CD86 expression by human monocytes infected with Col cl1.7, but not the Y strain [150]. An increase in the expression of CD80 by DCs infected with AQ1.7, MUTUM, and 2369 strains was also observed. However, this result was not observed in the 1849 strain [61]. These data suggest that the genetic variability found in the different *T. cruzi* strains may be important for driving the immune response and could also be related to the development of the different clinical forms of Chagas disease patients.

Conversely, the polarity of clinical manifestations could also be caused by the host background, mainly for its capacity to induce an effective immune response. A higher frequency of CD80^+^ monocytes in IND and CARD Chagas patients has been demonstrated after exposure to the Y strain of *T. cruzi* and the lower frequency of CD86^+^ monocytes in CARD patients, yet not in the IND form. Exposure of monocytes infected with *T. cruzi* to lymphocytes increased CTLA-4 expression in IND patients when compared to the control group [39].

On the other hand, a recent study also demonstrated an increase in CD80 expression in the total monocytes of IND and CARD patients and a reduction in CD86 expression in CARD individuals after being stimulated with cytoplasmic repetitive and flagellar repetitive *T. cruzi* antigens [151]. Moreover, a higher CD80 expression in classical monocytes of IND and CARD patients was seen, but only IND patients showed a greater CD86 expression in total, classical, intermediate, and non-classical monocytes as well as higher CD86 rather than CD80 expression [49]. In addition, the ligands of CD80 and CD86, CTLA-4 and CD28 molecules were evaluated on the total CD4^+^ T lymphocytes. Increased CD4^+^CTLA-4^+^ T lymphocyte frequency was observed in Chagas patients, mainly in the IND group. Moreover, an association between CD80 and CD28, and between CD86 and CTLA-4, was observed [49]. These results suggest that the CD80 molecule could be essential for the activation of lymphocytes after interaction with the CD28 receptor in Chagas disease patients regardless of the clinical form. CD86 is most strongly expressed in IND patients, and CD86/CTLA-4 engagement could be crucial for regulating the immune response and avoiding exacerbated inflammation in IND patients. The interaction between CD86 and CTLA-4 may be associated with Tregs differentiation in IND patients [49]. Therefore, while this interaction may contribute to the activation of Treg lymphocytes in IND patients and favor the formation of an immunoregulatory environment, Tregs activation in CARD patients could be mediated by the CD80 receptor. However, the activation of CD80-induced Treg lymphocytes in CARD patients appears to be inefficient in regulating the immune response, contributing to the formation of an uncontained inflammatory response as observed in the patients.

Although there are few works about the role of these costimulatory molecules in Chagas patients, it is essential to expand studies with murine models to confirm and consolidate the mechanisms of immune function. It was observed that CD28-deficient mice infected with the Tulahuen strain of *T. cruzi* had increased susceptibility to the infection. The blocking of CD80 and CD86 by monoclonal antibodies also exacerbated *T. cruzi* infection [152]. CD28 expression is reportedly seen to significantly decrease in T cells from Chagas patients [153]. CD28-T cells display a biased expression of the V-beta 5 T-cell receptor region, and the functional profile of CD28-expressing Vbeta5 region is distinct in patients with indeterminate and cardiac clinical forms [154]. CD28-deficient mice infected with Y or Colombian strains of *T. cruzi* showed high parasitemia and a lower survival rate. This receptor is essential to CD4^+^ T cell activation during *T. cruzi* infection [155] (Table 1).

## 3. Concluding Remarks

The heterogeneity of clinical manifestations of chronic Chagas disease has, until now, been one of the points to be unveiled by science. It is still unknown why some *T. cruzi*-infected patients develop cardiac and/or severe digestive clinical outcomes, while others do not present symptoms related to the pathology. As Chagas disease is a multifactorial pathology, different factors may be involved in its establishment, such as the *T. cruzi* strain, virulence factors, the route of infection, parasitic burden, and the host immune response. However, as in the chronic phase of Chagas disease few parasites can be found in the host tissue and bloodstream, the immune response may be the main factor that could be related to the polarity found between the different clinical forms of the pathology.

The co-stimulatory signals expressed by monocytes are essential to determining the magnitude of T cell response to the antigen, playing a crucial role in determining the regulation of subsequent adaptive immune response. However, little is known about the expression and function of these molecules in Chagas disease development, mainly on the ICOSL and ICOS receptors.

**Figure 1:**
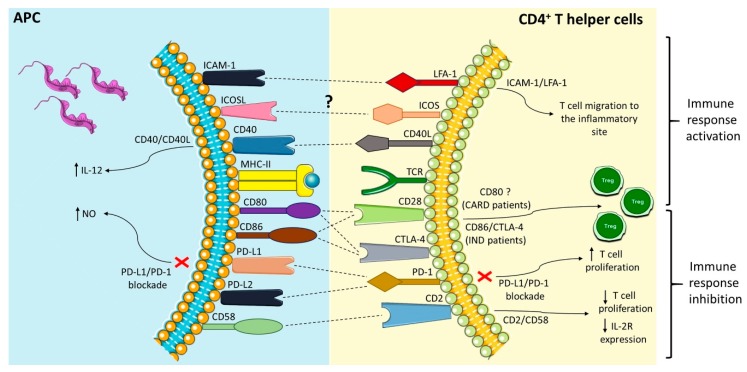
Interactions between co-stimulatory molecules present on professional antigen-presenting cell (APC) with CD4^+^ T helper cells and its possible role in Chagas disease. Dashed lines (---) represent the specific interaction between the receptors.

These co-stimulatory interactions (Figure 1) may result in the activation of different subsets of lymphocytes, which will lead to more or less efficient adaptive immune responses. Understanding these effects and the type of lymphocytic response activated can elucidate this gap in the pathogenesis of *T. cruzi* infection and the development of cardiac changes inherent to the parasite. Here, we have described important co-stimulatory mechanisms from the perspective of Chagas disease and its clinical manifestations during the chronic phase. However, it became clear that this is a field that needs to be explored, given the importance of the issue and the complexity of the mechanisms in Chagas disease.

## Figures and Tables

**Table 1:** The main co-stimulatory receptors and their possible roles in Chagas disease.

Co-Stimulatory Pathway	Cell Expression	Main Function	Main Findings in Chagas Disease	References
PD-1/PD-L1 or PD-L2	PD-1 (T cells, B cells, and myeloid cells); PD-L1 (B cells, DCs, and monocytes) and PD-L2 (DCs)	Inhibition of the activation of T cells	Different strains were able to induce the expression of PD-L1 on DC; PD-1 is highly expressed in lymphocytes found in the hearts of mice infected with *T. cruzi*, and its blockade increased the proliferative response by T cells and mice mortality; macrophages of infected mice treated with anti-PD-1 or anti-PD-L increased NO production.	[50,56,58,59,61,65,66]
ICOSL/ICOS	ICOS (T cell) and ICOSL (B cells, macrophages, and DCs)	Activation and differentiation of T cells	To this date, no study has shown a role of the ICOS/ICOSL pathway in *T. cruzi* infection.	[52,67,68,69,72]
CD40/CD40L	CD40 (APCs) and CD40L (T cell)	Activation of APCs and provision of co-stimulatory signals to T cells	C57BL/6 mice infected with *T. cruzi* showed high levels of CD40 by APCs; CD40/CD40L interaction on lymphocytes in the heart can induce IL-6 production and contribute to tissue inflammation; blockage of CD40 on human monocytes infected with *T. cruzi* causes 50% of the inhibition of the IL-12 expression.	[52,83,84,89,90,92]
CD95/CD95L	Several cells and T and B lymphocytes	Induction of apoptosis	CD95/CD95L engagement promotes apoptosis of CD4+ T cells co-cultivated with macrophages infected with *T. cruzi*; the deficiency of this receptor in modified mice or its blockage can increase IL-10 and IL-4 production.	[97,99,101,102]
CD2/CD58	CD2 (Red blood and T cells) and CD58 (APCs)	Activation of T cells	Co-cultures of human PBMCs infected with *T. cruzi* and stimulated with anti-CD2 antibodies showed a reduction in lymphocytes proliferative and IL-2R expression.	[106,107,108,119]
LFA-1/ICAM-1	LFA-1 (T cells) and ICAM-1 (Endothelium and APCs)	Adhesion, migration of T cells and antigen presentation	Leukocytes of the inflammatory infiltrate, circulating mononuclear cells and cardiomyocytes increased ICAM-1 expression in the acute infection with *T. cruzi*.	[109,120,121,122,123,127,128,129,131]
CD80-CD86/CD28-CTLA-4	CD80-CD86 (APCs) and CD28-CTLA-4 (CD4^+^ T cells)	CD80 or CD86 interaction with CD28 activates T cells, and with CTLA-4 inhibits the activation of lymphocytes	CD28-deficient mice and CD80/CD86 blockage increased susceptibility to *T. cruzi* infection; Chagas patients showed a higher frequency of CD80 monocytes; IND patients showed a high CD86 expression by monocytes and their subsets, CTLA-4 T CD4^+^ T lymphocytes frequency, and increased Treg proportion.	[39,49,135,137,138,139,151,152]

APCs—Antigen-presenting cells, DCs—Dendritic cells, IND—Indeterminate clinical form, NO—Nitric oxide, PBMC—Peripheral blood mononuclear cell, *T. cruzi*—*Trypanosoma cruzi*.

## References

[1] Álvares J.M., Fonseca R., Silva H.B., Marinho C.R.F., Bortoluci K.R., Sardinha L.R., Epiphanio S., Lima M.R.I. (2014). Chagas disease: Still many unsolved issues. Mediat. Inflamm..

[2] World Health Organization (WHO) (2015). Investing to Overcome the Global Impact of Neglected Tropical Diseases: Third WHO Report on Neglected Diseases 2015.

[3] Pérez-Molina J.A., Molina I. (2017). Chagas disease. Seminar.

[4] World Health Organization (WHO) Chagas Disease (American Trypanosomiasis). http://www.who.int/chagas/disease/en/.

[5] Dias J.C.P., Ramos A.N., Gontijo E.D., Luquetti A., Shikanai-Yasusa M.A., Coura J.R., Torres R.M., Melo J.R.C., Alemida E.A., Oliveira W. (2016). Consenso brasileiro em doença de Chagas. Epidemiol. Serv. Saúde.

[6] Yamagata Y., Nakagawa J. (2006). Control of Chagas disease. Adv. Parasitol..

[7] Iniciativa de Salud del Cono Sur, Incosur (2005). XIV Reunión de la Comisión Intergubernamental del Cono Sur para la Eliminación de Triatoma Infestans y la Interrupción de la Transmisión de la Tripanosomiasis Transfusional y Curso de Diagnóstico, Manejo y Tratamiento de Enfermedad de Chagas.

[8] Schmunis G.A., Yadon Z.E. (2010). Chagas disease: A latin american health problem becoming a world health problem. Acta Trop..

[9] Coura J.R., Viñas P.A., Junqueira A.C.V. (2014). Ecoepidemiology, short history and control of Chagas disease in the endemic countries and the new challenge for non-endemic countries. Mem. Inst. Oswaldo Cruz.

[10] Ayo C.M., Dalalio M.M.O., Visentainer J.E.L., Reis P.G., Sippert E.A., Jarduli L.R., Alves H.V., Sell A.M. (2013). Genetic Susceptibility to Chagas disease: An overview about the infection and about the association between disease and the immune response genes. Biomed. Res. Int..

[11] Andrade D.V., Gollob K.J., Dutra W.O. (2014). Acute Chagas disease: New global challenges for an old Neglected disease. PLoS Negl. Trop. Dis..

[12] Prata A. (1990). Classificação da infecção chagásica no homem. Rev. Soc. Bras. Med. Trop..

[13] Brener Z., Gazzinelli R.T. (1997). Immunological control of *Trypanosoma cruzi* infection and pathogenesis of Chagas’ disease. Int. Arch. Allergy Immunol..

[14] Teixeira A.R.L., Hecht M.M., Guimaro M.C., Sousa A.O., Nitz N. (2011). Pathogenesis of Chagas’ disease: Parasite persistence and autoimmunity. Clin. Microbiol. Rev..

[15] Domingues C.S., Hardoim D.J., Souza C.S.F., Cardoso F.O., Mendes V.G., Previtalli-Silva H., Abreu-Silva A.L., Pelajo-Machado M., Costa S.C.G., Calabrese K.S. (2015). Oral outbreak of Chagas disease in Santa Catarina, Brazil: Experimental evaluation of a patient’s strain. PLoS ONE.

[16] Dario M.A., Rodrigues M.S., Barros J.H.S., Xavier S.C.C., D’Andrea O.S., Roque A.L.R., Jansen A.M. (2016). Ecological scenario and *Trypanosoma cruzi* DTU characterization of a fatal acute Chagas disease case transmitted orally (Espírito Santo state, Brazil). Parasit. Vectors.

[17] Ministério da Saúde Doença de Chagas Aguda-Casos Confirmados Notificados no Sistema de Informação de Agravos de Notificação-Brasil. http://tabnet.datasus.gov.br/cgi/deftohtm.exe?sinannet/cnv/chagasbr.def.

[18] Ribeiro A.L.P., Nunes M.P., Teixeira M.M., Rocha M.O. (2012). Diagnosis and management of Chagas disease and cardiomyopathy. Nat. Rev. Cardiol..

[19] Steverding D. (2014). The history of Chagas disease. Parasit. Vectors.

[20] Ribeiro A.L.P., Rocha M.O. (1998). Forma indeterminada da doença de Chagas: Considerações acerca do diagnóstico e do prognóstico. Rev. Soc. Bras. Med. Trop..

[21] Mathews J.C. (1973). Valor de la Prueba de Esfuerzo Graduado (Ergometria) para Determinar la Capacidad Laboral del Cardiópata Chagásico Crônico. Ph.D. Thesis.

[22] Macêdo V. (1999). Indeterminate form of Chagas disease. Mem. Inst. Oswaldo Cruz.

[23] Nogueira L.G., Santos R.H.B., Fiorelli A.I., Mairena E.C., Benvenuti L.A., Bocchi E.A., Stolf N.A., Kalil J., Cunha-Neto E. (2014). Myocardial gene expression of T-bet, GATA-3, Ror-*γ*t, FoxP3, and hallmark cytokines in chronic Chagas disease cardiomyopathy: An essentially unopposed TH1-Type Response. Mediat. Inflamm..

[24] Malik L.H., Singh G.D., Amsterdam E.A. (2015). Chagas heart disease: An update. Am. J. Med..

[25] Andrade Z.A. (1985). A Patologia da doença de Chagas no Homem. Ann. Soc. Belg. Med. Trop..

[26] Guedes P.M.M., Veloso V.M., Afonso L.C.C., Caliari M.V., Carneiro C.M., Diniz L.F., Marques-da-Silva E.A., Caldas I.S., Matta M.A.V., Souza S.M. (2009). Development of chronic cardiomyopathy in canine Chagas disease correlates with high IFN-γ, TNF-α, and low IL-10 production during the acute infection phase. Vet. Immunol. Immunopathol..

[27] Frade A.F., Teixeira P.C., Ianni B.M., Pissetti C.W., Saba B., Wang L.H.T., Kuramoto A., Nogueira L.G., Buck P., Dias F. (2013). Polymorphism in the alpha cardiac muscle actin 1 gene is associated to susceptibility to chronic inflammatory cardiomyopathy. PLoS ONE.

[28] Stanaway J.D., Roth G. (2015). The burden of Chagas disease: Estimates and challenges. Glob. Heart.

[29] Brener Z., Andrade Z.A., Koogan G. (1979). *Trypanosoma cruzi* e doença de Chagas. Epidemiologia.

[30] Rezende J.M., Moreira H., Castro L.P., Coelho L.G.V. (2004). Forma digestiva da doença de Chagas. Gastroenterologia.

[31] Malik L.H., Singh G.D., Amsterdam E.A. (2015). The epidemiology, clinical manifestations, and management of chagas heart disease. Clin. Cardiol..

[32] Gomes J.A.S., Bahia-Oliveira L.M.G., Rocha M.O.C., Martins-Filho O.A., Gazzinelli G., Correa-Oliveira R. (2003). Evidence that development of severe cardiomyopathy in human Chagas’ disease is due to a Th1-specific immune response. Infect. Immun..

[33] Savino W., Villa-Verdea D.M.S., Mendes-da-Cruza D.A., Silva-Monteiro E., Perez A.R., Aoki M.P., Bottasso O., Guiñazú N., Silva-Barbosa S.D., Gea S. (2007). Cytokines and cell adhesion receptors in the regulation of immunity to *Trypanosoma cruzi*. Cytokine Growth Factor Rev..

[34] Chaves A.T., Gomes J.A.S., Fiuza J.A., Carvalho A.T., Ferreira K.S., Fares R.C.G., Guimarães P.H.G., Fagundes S.E.M., Morato M.J., Fujiwara R.T. (2016). Immunoregulatory mechanisms in Chagas disease: Modulation of apoptosis in T-cell mediated immune responses. BMC Infect. Dis..

[35] Gomes J.A.S., Molica A.M., Keesen T.S.L., Morato M.J.F., De Araújo F.F., Fares R.C.G., Fiuza J.Á., Chaves A.T., Pinheiro V., Nunes M.C.P. (2013). Inflammatory mediators from monocytes down-regulate cellular proliferation and enhance cytokines production in patients with Polar clinical forms of Chagas disease. Hum. Immunol..

[36] Junqueira C., Caetano B., Bartholomeu D.C., Melo M.B., Ropert C., Rodrigues M.M., Gazzinelli R.T. (2010). The endless race between *Trypanosoma cruzi* and host immunity: Lessons for and beyond Chagas disease. Expert Rev. Mol. Med..

[37] Gravina H.D., Antonelli L., Gazzinelli R.T., Ropert C. (2013). Differential use of TLR2 and TLR9 in the regulation of immune responses during the infection with *Trypanosoma cruzi*. PLoS ONE.

[38] Bartholomeu D.C., Ropert C., Melo M.B., Parroche P., Junqueira C.F., Teixeira S.M., Sirois C., Kasperkovitz P., Knetter C.F., Lien E. (2008). Recruitment and endo-lysosomal activation of TLR9 in dendritic cells infected with *Trypanosoma cruzi*. J. Immunol..

[39] Souza P.E.A., Rocha M.O.C., Menezes C.A.S., Coelho J.S., Chaves A.C.L., Gollob K.J., Dutra W.O. (2007). *Trypanosoma cruzi* infection induces differential modulation of costimulatory molecules and cytokines by monocytes and T cells from patients with indeterminate and cardiac Chagas’ disease. Infect. Immun..

[40] Cunha-Neto E., Nogueira L.G., Teixeira P.C., Ramasawmy R., Drigo S.A., Goldberg A.C., Fonseca S.G., Bilate A.M., Kalil J. (2009). Immunological and non-immunological effects of cytokines and chemokines in the pathogenesis of chronic Chagas disease cardiomyopathy. Mem. Inst. Oswaldo Cruz.

[41] Rodrigues D.B.R., Reis M.A., Romano A., Pereira S.A.L., Teixeira V.P.A., Junior S.T., Rodrigues V. (2012). In situ expression of regulatory cytokines by heart inflammatory cells in Chagas’ disease patients with heart failure. Clin. Dev. Immunol..

[42] Basso B. (2013). Modulation of immune response in experimental Chagas disease. World J. Exp. Med..

[43] De Araújo F.F., Corrêa-Oliveira R., Rocha M.O.C., Chaves A.T., Fiuza J.A., Fares R.C.G., Ferreira K.S., Nunes M.C.P., Keesen T.S., Damasio M.P.S. (2012). Foxp3^+^CD25^high^CD4^+^ regulatory T cells from indeterminate patients with Chagas disease can suppress the effector cells and cytokines and reveal altered correlations with disease severity. Immunobiology.

[44] Melo A.S., Lorena V.M.B., Braz S.C.M., Docenab C., Gomes Y.M. (2012). IL-10 and IFN-γ gene expression in chronic Chagas disease patients after in vitro stimulation with recombinant antigens of *Trypanosoma cruzi*. Cytokine.

[45] Magalhães L.M.D., Villani F.N.A., Nunes M.C.P., Gollob K.J., Rocha M.O.C., Dutra W.O. (2013). High interleukin 17 expression is correlated with better cardiac function in human Chagas disease. J. Infect. Dis..

[46] Dutra W.O., Menezes C.A.S., Magalhães L.M.D., Gollob K.J. (2014). Immunoregulatory networks in human Chagas disease. Parasite Immunol..

[47] Souza P.E.A., Rocha M.O.C., Rocha-Vieira E., Menezes C.A.S., Chaves A.C.L., Gollob K.J., Dutra W.O. (2004). Monocytes from patients with indeterminate and cardiac forms of Chagas’ disease display distinct phenotypic and functional characteristics associated with morbidity. Infect. Immun..

[48] Dutra W.O., Menezes C.A., Villani F.N., da Costa G.C., da Silveira A.B., Reis D.D., Gollob K.J. (2009). Cellular and genetic mechanisms involved in the generation of protective and pathogenic immune responses in human Chagas disease. Mem. Inst. Oswaldo Cruz.

[49] Pinto B.F., Medeiros N.I., Teixeira-Carvalho A., Eloi-Santos S.M., Fontes-Cal T.C.M., Rocha D.A., Dutra W.O., Correa-Oliveira R., Gomes J.A.S. (2018). CD86 expression by monocytes influences an immunomodulatory profile in asymptomatic patients with chronic Chagas disease. Front. Immunol..

[50] Sharpe A.R., Freeman G.J. (2002). The B7–CD28 superfamily. Nat. Immunol..

[51] Salomon B., Bluestone J.A. (2001). Complexities of CD28/B7: CTLA-4 costimulatory pathways in autoimmunity and transplantation. Annu. Rev. Immunol..

[52] Sharpe A.R. (2009). Mechanisms of costimulation. Immunol. Rev..

[53] Sansom D.M. (2000). CD28, CTLA-4 and their ligands: Who does what and to whom?. Immunology.

[54] Nishimura H., Nose M., Hiai H., Minato N., Honjo T. (1999). Development of lupus-like autoimmune diseases by disruption of the PD-1 gene encoding an ITIM motif-carrying immunoreceptor. Immunity.

[55] Nishimura H., Honjo T. (2001). PD-1: An inhibitory immunoreceptor involved in peripheral tolerance. Trends Immunol..

[56] Schachtele S.J., Hu S., Sheng W.S., Mutnal M.B., Lokensgard J.R. (2014). Glial cells suppress postencephalitic CD8^+^ T lymphocytes through PD-L1. Glia.

[57] Guo Y., Walsh A.M., Canavan M., Wechalekar M.D., Cole S., Yin X., Scott B., Loza M., Orr C., McGarry T. (2018). Immune checkpoint inhibitor PD-1 pathway is down-regulated in synovium at various stages of rheumatoid arthritis disease progression. PLoS ONE.

[58] Freeman G.J., Long A.J., Iwai Y., Bourque K., Chernova T., Nishimura H., Fitz L.J., Malenkovich N., Okazaki T., Byrne M.C. (2000). Engagement of the PD-1 immunoinhibitory receptor by a novel B7 family member leads to negative regulation of lymphocyte activation. J. Exp. Med..

[59] Agata Y., Kawasaki A., Nishimura H., Ishida Y., Tsubata T., Yagita H., Honjo T. (1996). Expression of the PD-1 antigen on the surface of stimulated mouse T and B lymphocytes. Int. Immunol..

[60] Stempin C.C., Marquez J.D.R., Ana Y., Cerban F.M. (2017). GRAIL and Otubain-1 are related to T cell hyporesponsiveness during *Trypanosoma cruzi* infection. PLoS Negl. Trop. Dis..

[61] Da Costa T.A., Silva M.V., Mendes M.T., Carvalho-Costa T.M., Batista L.R., Lages-Silva E., Rodrigues V., Oliveira C.J., Ramirez L.E. (2014). Immunomodulation by *Trypanosoma cruzi*: Toward understanding the association of dendritic cells with infecting TcI and TcII populations. J. Immunol. Res..

[62] Dias F.C., Medina T.S., Mendes-Junior C.T., Dantas R.O., Pissetti C.W., Junior V.R., Dellalibera-Joviliano R., Marin-Neto J.A., Gutierrez F.R.S., Moreau P. (2013). Polymorphic sites at the immunoregulatory CTLA-4 gene are associated with chronic Chagas disease and its clinical manifestations. PLoS ONE.

[63] Lasso P., Mateus J., Pavía P., Rosas F., Roa N., Thomas M.C., López M.C., González J.M., Puerta C.J., Cuéllar A. (2015). Inhibitory receptor expression on CD8^+^ T cells is linked to functional responses against *Trypanosoma cruzi* antigens in chronic chagasic patients. J. Immunol..

[64] Borges D.C., Araújo N.M., Cardoso C.R., Chica J.E.L. (2012). Different parasite inocula determine the modulation of the immune response and outcome of experimental *Trypanosoma cruzi* infection. Immunology.

[65] Gutierrez F.R.S., Mariano F.S., Oliveira C.J.F., Pavanelli W.R., Guedes P.M.M., Silva G.K., Campanelli A.P., Milanezi C.M., Azuma M., Honjo T. (2011). Regulation of *Trypanosoma cruzi*-induced myocarditis by programmed death cell receptor. Infect. Immun..

[66] Dulgerian L.R., Garrido V.V., Stempin C.C., Cerbán F.M. (2011). Programmed death ligand 2 regulates arginase induction and modifies *Trypanosoma cruzi* survival in macrophages during murine experimental infection. Immunology.

[67] Cassiano G.C., Santos E.J.M., Maia M.H.T., Furini A.C., Storti-Melo L.M., Tomaz F.M.B., Trindade P.C.A., Capobianco M.P., Amador M.A.T., Viana G.M.R. (2015). Impact of population admixture on the distribution of immune response costimulatory genes polymorphisms in a Brazilian population. Hum. Immunol..

[68] Amatore F., Gorvel L., Olive D. (2018). Inducible Co-Stimulator (ICOS) as a potential therapeutic target for anti-cancer therapy. Expert Opin. Ther. Target..

[69] Kovaleva M., Johnson K., Steven J., Barelle C.J., Porter A. (2017). Therapeutic potential of shark Anti-ICOSL VNAR domains is exemplified in a murine model of autoimmune non-infectious uveitis. Front. Immunol..

[70] Brodie D., Collins A.V., Iaboni A., Fennelly J.A., Sparks L.M., Xu X.-N., van der Merwe P.A., Davis S.J. (2000). LICOS, a primordial costimulatory ligand?. Curr. Biol..

[71] Yao S., Zhu Y., Chen L. (2013). Advances in targeting cell surface signalling molecules for immune modulation. Nat. Rev. Drug Discov..

[72] Dong C., Juedes A.E., Temann U.A., Shresta S., Allison J.P., Ruddle N.H., Flavell R.A. (2001). ICOS co-stimulatory receptor is essential for T-cell activation and function. Nature.

[73] Villegas E.N., Elloso M.M., Reichmann G., Peach R., Hunter C.A. (1999). Role of CD28 in the generation of effector and memory responses required for resistance to *Toxoplasma gondii*. J. Immunol..

[74] Villegas E.N., Lieberman L.A., Mason N., Blass S.L., Zediak V.P., Peach R., Horan T., Yoshinaga S., Hunter C.A. (2002). A role for inducible costimulator protein in the CD28-independent mechanism of resistance to *Toxoplasma gondii*. J. Immunol..

[75] Greenwald R.J., McAdam A.J., Woude D.V., Satoskar A.R., Sharpe A.H. (2002). Cutting edge: Inducible costimulator protein regulates both Th1 and Th2 responses to cutaneous leishmaniasis. J. Immunol..

[76] Dempsey P.W., Doyle S.E., He J.Q., Cheng G. (2003). The signaling adaptors and pathways activated by TNF superfamily. Cytokine Growth Factor Rev..

[77] Locksley R.M., Killeen N., Lenardo M.J. (2001). The TNF and TNF receptor superfamilies: Integrating mammalian biology. Cell.

[78] Chung J.Y., Park Y.C., Ye H., Wu H. (2002). All TRAFs are not created equal: Common and distinct molecular mechanisms of TRAF-mediated signal transduction. J. Cell Sci..

[79] Watts T.H. (2005). TNF/TNFR family members in costimulation of T cell responses. Annu. Rev. Immunol..

[80] Ward-Kavanagh L., Lin W.W., Šedý J.S., Ware C.F. (2016). The TNF receptor superfamily in costimulating and coinhibitory responses. Immunity.

[81] Bodmer J.L., Schneider P., Tschopp J. (2002). The molecular architecture of the TNF superfamily. Trends Biochem. Sci..

[82] Vanarsdale T.L., Vanarsdale S.L., Force W.R., Walter B.N., Mosialos G., Kieff E., Reed J.C., Ware C.F. (1997). Lymphotoxin-β receptor signaling complex: Role of tumor necrosis factor receptor-associated factor 3 recruitment in cell death and activation of nuclear factor kβ. Immunology.

[83] Kollias G., Kontoyiannis D. (2002). Role of TNF/TNFR in autoimmunity: Specific TNF receptor blockade may be advantageous to anti-TNF treatments. Cytokine Growth Factor Rev..

[84] Croft M. (2003). Co-stimulatory members of the TNFR family: Keys to effective T-cell immunity?. Nat. Rev. Immunol..

[85] Shimozato O., Takeda K., Yagita H., Okumura K. (1999). Expression of CD30 ligand (CD153) on murine activated T cells. Biochem. Biophys. Res. Commun..

[86] Gramaglia I., Cooper D., Miner K.T., Kwon B.S., Croft M. (2000). Co-stimulation of antigen-specific CD4 T cells by 4-1BB ligand. Eur. J. Immunol..

[87] Martínez Gómez J.M., Koh V.H., Yan B., Lin W., Ang M.L., Rahim S.Z., Pethe K., Schwarz H., Alonso S. (2014). Role of the CD137 ligand (CD137L) signaling pathway during *Mycobacterium tuberculosis* infection. Immunobiology.

[88] Van de Ven K., Borst J. (2015). Targeting the T-cell co-stimulatory CD27/CD70 pathway in cancer immunotherapy: Rationale and potential. Immunotherapy.

[89] Planelles L., Thomas M.C., Marañón C., Morell M., López M.C. (2003). Differential CD86 and CD40 co-stimulatory molecules and cytokine expression pattern induced by *Trypanosoma cruzi* in APCs from resistant or susceptible mice. Clin. Exp. Immunol..

[90] Ayala M.A., Casasco A., González M., Postan M., Corral R.S., Petray P.B. (2016). *Trypanosoma cruzi* infection induces the expression of CD40 in murine cardiomyocytes favoring CD40 ligation-dependent production of cardiopathogenic IL-6. Parasitol. Res..

[91] Chaussabel D., Jacobs F., de Jonge J., de Veerman M., Carlier Y., Thielemans K., Goldman M., Vray B. (1999). CD40 ligation prevents *Trypanosoma cruzi* infection through interleukin-12 upregulation. Infect. Immun..

[92] Abel L.C., Ferreira L.R., Cunha Navarro I., Baron M.A., Kalil J., Gazzinelli R.T., Rizzo L.V., Cunha-Neto E. (2014). Induction of IL-12 production in human peripheral monocytes by *Trypanosoma cruzi* is mediated by glycosylphosphatidylinositol-anchored mucin-like glycoproteins and potentiated by IFN-γ and CD40-CD40L interactions. Mediat. Inflamm..

[93] Martin S., Agarwal R., Murugaiyan G., Saha B. (2010). CD40 expression levels modulate regulatory T cells in *Leishmania donovani* infection. J. Immunol..

[94] Kamanaka M., Yu P., Yasui T., Yoshida K., Kawabe T., Horii T., Kishimoto T., Kikutani H. (1996). Protective role of CD40 in *Leishmania major* infection at two distinct phases of cell-mediated immunity. Immunity.

[95] Tuladhar R., Natarajan G., Satoskar A.R. (2011). Role of co-stimulation in Leishmaniasis. Int. J. Biol. Sci..

[96] Campbell K.A., Ovendale P.J., Kennedy M.K., Fanslow W.C., Reed S.G., Maliszewski C.R. (1996). CD40 ligand is required for protective cell-mediated immunity to *Leishmania major*. Immunity.

[97] Martins G.A., Vieira L.Q., Cunha F.Q., Silva J.S. (1999). Gamma interferon modulates CD95 (Fas) and CD95 ligand (Fas-L) expression and nitric oxide-induced apoptosis during the acute phase of *Trypanosoma cruzi* infection: A possible role in immune response control. Infect. Immun..

[98] Gustafsson A.B., Gottlieb R.A. (2003). Mechanisms of apoptosis in the heart. J. Clin. Immunol..

[99] Lula J.F., Rocha M.O., Nunes M.C.P., Ribeiro A.L., Teixeira M.M., Bahia M.T., Talvani A. (2009). Plasma concentrations of tumour necrosis factor-alpha, tumour necrosis factor-related apoptosis-inducing ligand, and FasLigand/CD95L in patients with Chagas cardiomyopathy correlate with left ventricular dysfunction. Eur. J. Heart Fail..

[100] Tostes S., Rocha-Rodrigues D.B., de Araujo Pereira G., Rodrigues V. (2005). Myocardiocyte apoptosis in heart failure in chronic Chagas’ disease. Int. J. Cardiol..

[101] Guillermo L.V., Silva E.M., Ribeiro-Gomes F.L., De Meis J., Pereira W.F., Yagita H., DosReis G.A., Lopes M.F. (2007). The Fas death pathway controls coordinated expansions of type 1 CD8 and type 2 CD4 T cells in *Trypanosoma cruzi* infection. J. Leukocyte Biol..

[102] Lopes M.F., Nunes M.P., Henriques-Pons A., Giese N., Morse H.C., Davidson W.F., Araújo-Jorge T.C., Dos Reis G.A. (1999). Increased susceptibility of Fas ligand-deficient gld mice to *Trypanosoma cruzi* infection due to a Th2-biased host immune response. Eur. J. Immunol..

[103] Keesen T.S., Gomes J.A., Fares R.C., de Araújo F.F., Ferreira K.S., Chaves A.T., Rocha M.O., Correa-Oliveira R. (2012). Characterization of CD4^+^ cytotoxic lymphocytes and apoptosis markers induced by *Trypanossoma cruzi* infection. Scand. J. Immunol..

[104] Killeen N., Stuart S.G., Littman D.R. (1992). Development and function of T cells in mice with a disrupted CD2 gene. EMBO J..

[105] Davis S.J., van der Merwe P.A. (1996). The structure and ligand interactions of CD2: Implications for T-cell function. Immunol. Today.

[106] Hünig T., Tiefenthaler G., Meyer zum Büschenfelde K.H., Meuer S.C. (1987). Alternative pathway activation of T cells by binding of CD2 to its cell-surface ligand. Nature.

[107] Green J.M., Karpitskiy V., Kimzey S.L., Shaw A.S. (2000). Coordinate regulation of T cell activation by CD2 and CD28. J. Immunol..

[108] Skånland S.S., Moltu K., Berge T., Aandahl E.M., Taskén K. (2014). T-cell co-stimulation through the CD2 and CD28 co-receptors induces distinct signalling responses. Biochem. J..

[109] Wingren A.G., Parra E., Varga M., Kalland T., Sjogren H.O., Hedlund G., Dohlsten M. (1995). T Cell activation pathways: B7, LFA-3, and ICAM-1 shape unique T cell profiles. Crit. Rev. Immunol..

[110] Croft M., Dubey C. (1997). Accessory molecule and costimulation requirements for CD4 T cell response. Crit. Rev. Immunol..

[111] Shahinian A., Pfeffer K., Lee K.P., Kündig T.M., Kishihara K., Wakeham A., Kawai K., Ohashi P.S., Thompson C.B., Mak T.W. (1993). Differential T cell costimulatory requirements in CD28-deficient mice. Science.

[112] Liu L.L., Landskron J., Ask E.H., Enqvist M., Sohlberg E., Traherne J.A., Hammer Q., Goodridge J.P., Larsson S., Jayaraman J. (2016). Critical role of CD2 co-stimulation in adaptive natural killer cell responses revealed in NKG2C-deficient humans. Cell Rep..

[113] Murali A.R., Chandra S., Stewart Z., Blazar B.R., Farooq U., Ince M.N., Dunkelberg J. (2016). Graft versus host disease after liver transplantation in adults: A case series, review of literature, and an approach to management. Transplantation.

[114] Koumakis E., Bouaziz M., Dieudé P., Cauvet A., Ruiz B., Airò P., Cusi D., Matucci-Cerinic M., Salvi E., Cuomo G. (2016). A candidate gene study identifies a haplotype of CD2 as novel susceptibility factor for systemic sclerosis. Clin. Exp. Rheumatol..

[115] Sharma T.L., Yeaney G.A., Soltanzadeh P., Li Y., Cotta C.V. (2017). Intravascular T-cell lymphoma: A rare, poorly characterized entity with cytotoxic phenotype. Neuropathology.

[116] Bolduan S., Koppensteiner H., Businger R., Rebensburg S., Kunze C., Brack-Werner R., Draenert R., Schindler M. (2017). T cells with low CD2 levels express reduced restriction factors and are preferentially infected in therapy naïve chronic HIV-1 patients. J. Int. AIDS Soc..

[117] Tsitsikov E., Harris M.H., Silverman L.B., Sallan S.E., Weinberg O.K. (2018). Role of CD81 and CD58 in minimal residual disease detection in pediatric B lymphoblastic leukemia. Int. J. Lab. Hematol..

[118] El Menshawy N., Eissa M., Abdeen H.M., Elkhamisy E.M., Joseph N. (2018). CD58; leucocyte function adhesion-3 (LFA-3) could be used as a differentiating marker between immune and non-immune thyroid disorders. Comp. Clin. Pathol..

[119] Beltz L.A., Kierszenbaum F., Sztein M.B. (1990). *Trypanosoma cruzi*-induced suppression of human peripheral blood lymphocytes activated via the alternative (CD2) pathway. Infect. Immun..

[120] Reis D.D., Jones E.M., Tostes S., Lopes E.R., Chapadeiro E., Gazzinelli G., Colley D.G., McCurley T.L. (1993). Expression of major histocompatibility complex antigens and adhesion molecules in hearts of patients with chronic Chagas’ disease. Am. J. Trop. Med. Hyg..

[121] Hogg N., Laschinger M., Giles K., McDowall A. (2003). T-cell integrins: More than just sticking points. J. Cell. Sci..

[122] Abdullahi M., Olotu F.A., Soliman M.E. (2018). Allosteric inhibition abrogates dysregulated LFA-1 activation: Structural insight into mechanisms of diminished immunologic disease. Comput. Biol. Chem..

[123] Walling B.L., Kim M. (2018). LFA-1 in T cell migration and differentiation. Front. Immunol..

[124] Wang Y., Kai H., Chang F., Shibata K., Tahara-Hanaoka S., Honda S., Shibuya A., Shibuya K. (2007). A critical role of LFA-1 in the development of Th17 cells and induction of experimental autoimmune encephalomyelytis. Biochem. Biophys. Res. Commun..

[125] Verma N.K., Dempsey E., Long A., Davies A., Barry S.P., Fallon P.G., Volkov Y., Kelleher D. (2012). Leukocyte function-associated antigen-1/intercellular adhesion molecule-1 interaction induces a novel genetic signature resulting in T-cells refractory to transforming growth factor-β signaling. J. Biol. Chem..

[126] Verma N.K., Fazil M.H.U.T., Ong S.T., Chalasani M.L.S., Low J.H., Kottaiswamy A., Praseetha P., Kizhakeyil A., Kumar S., Panda A.K. (2016). LFA-1/ICAM-1 ligation in human T cells promotes Th1 polarization through a GSK3β signaling-dependent notch pathway. J. Immunol..

[127] Zhang L., Tarleton R.L. (1996). Persistent production of inflammatory and anti-inflammatory cytokines and associated MHC and adhesion molecule expression at the site of infection and disease in experimental *Trypanosoma cruzi* infections. Exp. Parasitol..

[128] Dos Santos P.V., Roffê E., Santiago H.C., Torres R.A., Marino A.P., Paiva C.N., Silva A.A., Gazzinelli R.T., Lannes-Vieira J. (2001). Prevalence of CD8 (+) alpha beta T cells in *Trypanosoma cruzi*-elicited myocarditis is associated with acquisition of CD62L(Low)LFA-1(High)VLA-4(High) activation phenotype and expression of IFN-gamma-inducible adhesion and chemoattractant molecules. Microbes Infect..

[129] Marino A.P., Azevedo M.I., Lannes-Vieira J. (2003). Differential expression of adhesion molecules shaping the T-cell subset prevalence during the early phase of autoimmune and *Trypanosoma cruzi*-elicited myocarditis. Mem. Inst. Oswaldo Cruz.

[130] Ferreira C.P., Cariste L.M., Santos Virgílio F.D., Moraschi B.F., Monteiro C.B., Vieira Machado A.M., Gazzinelli R.T., Bruna-Romero O., Menin Ruiz P.L., Ribeiro D.A. (2017). LFA-1 mediates cytotoxicity and tissue migration of specific CD8^+^ T cells after heterologous prime-boost vaccination against *Trypanosoma cruzi* infection. Front. Immunol..

[131] Laucella S., Salcedo R., Castaños-Velez E., Riarte A., De Titto E.H., Patarroyo M., Orn A., Rottenberg M.E. (1996). Increased expression and secretion of ICAM-1 during experimental infection with *Trypanosoma cruzi*. Parasite Immunol..

[132] Linsley P.S., Ledbetter J.A. (1993). The role of the CD28 receptor during T-cell responses to antigen. Annu. Rev. Immunol..

[133] Jenkins M.K., Schwartz R.H. (1987). Antigen presentation by chemically modified splenocytes induces antigen-specific T cell unresponsiveness in vitro and in vivo. J. Exp. Med..

[134] Van Gool S.W., Vandenberghe P., de Boer M., Ceuppens J.L. (1996). CD80, CD86 and CD40 provide accessory signals in a multiple-step T-cell activation model. Immunol. Rev..

[135] Ziller C., Stoeckel F., Boon L., Haegel-Kronenberger H. (2002). Transient blocking of both B7.1 (CD80) and B7.2 (CD86) in addition to CD40–CD40L interaction fully abrogates the immune response following systemic injection of adenovirus vector. Gene Ther..

[136] Linsley P.S., Bradshaw J., Greene J., Peach R., Bennett K.L., Mittler R.S. (1996). Intracellular trafficking of CTLA-4 and focal localization towards sites of TCR engagement. Immunity.

[137] Linsley P.S., Greene J.L., Brady W., Bajorath J., Ledbetter J.A., Peach R. (1994). Human B7-1 (CD80) and B7-2 (CD86) bind with similar avidities but distinct kinetics to CD28 and CTLA-4 receptors. Immunity.

[138] Lenschow D.J., Su G.H., Zuckerman L.A., Nabavi N., Jellis C.L., Gray G.S., Miller J., Bluestone J.A. (1993). Expression and functional significance of an additional ligand for CTLA-4. Proc. Natl. Acad. Sci. USA.

[139] Hathcock K.S., Laszlo G., Pucillo S.C., Linsley P., Hodes R.J. (1994). Comparative analysis of B7-1 and B7-2 costimulatory ligands: Expression and function. J. Exp. Med..

[140] Sigal L.J., Reiser H., Rock K.L. (1998). The role of B7-1 and B7-2 costimulation for the generation of CTL responses in vivo. J. Immunol..

[141] Waterhouse P., Penninger J.M., Timms E., Wakeham A., Shahinian A., Lee K.P., Thompson C.B., Griesser H., Mak T.W. (1995). Lymphoproliferative disorders with early lethality in mice deficient in Ctla-4. Science.

[142] Green J.M., Noel P.J., Sperling A.I., Walunas T.L., Gray G.S., Bluestone J.A., Thompson C.B. (1994). Absence of B7-dependent responses in CD-28 deficient mice. Immunity.

[143] Yu X., Fournier S., Allison J.P., Sharpe A.H., Hodes R.J. (2000). The role of B7 costimulation in CD4/CD8 T cell homeostasis. J. Immunol..

[144] June C.H., Ledbetter J.A., Linsley P.S., Thompson C.B. (1990). Role of the CD28 receptor in T-cell activation. Immunol. Today.

[145] Van Gool S.W., Vermeiren J., Rafiq K., Lorr K., de Boer M., Ceuppens J.L. (1999). Blocking CD40–CD154 and CD80/CD86–CD28 interactions during primary allogeneic stimulation results in T cell anergy and high IL-10 production. Eur. J. Immunol..

[146] Zheng Y., Manzotti C.N., Liu M., Burke F., Mead K.I., Sansom D.M. (2004). CD86 and CD80 differentially modulate the suppressive function of human regulatory T cells. J. Immunol..

[147] Qureshi O.S., Zheng Y., Nakamura K., Attridge K., Manzotti C., Schmidt E.M., Baker J., Jeffery L.E., Kaur S., Briggs Z. (2011). Trans-endocytosis of CD80 and CD86: A molecular basis for the cell-extrinsic function of CTLA-4. Science.

[148] Gardner D., Jeffery L.E., Sansom D.M. (2014). Understanding the CD28/CTLA-4 (CD152) pathway and its implications for costimulatory blockade. Am. J. Transplant..

[149] Flörcken A., Johannsen M., Nguyen-Hoai T., Gerhardt A., Miller K., Dörken B., Pezzutto A., Westermann J., Jöhrens K. (2017). Immunomodulatory molecules in renal cell cancer: CD80 and CD86 are expressed on tumor cells. Int. J. Clin. Exp. Pathol..

[150] Magalhães L.M.D., Viana A., Chiari E., Galvao L.M.C., Gollob K.J., Dutra W.O. (2015). Differential activation of human monocytes and lymphocytes by distinct strains of *Trypanosoma cruzi*. PLoS Negl. Trop. Dis..

[151] Soares A.K.D., Neves P.A.F., Cavalcanti M., Marinho S.M., de Oliveira W., de Souza J.R., Barros de Lorena V.M., Gomes Y.M. (2016). Expression of co-stimulatory molecules CD80 and CD86 is altered in CD14^+^HLA-DR^+^ monocytes from patients with Chagas disease following induction by *Trypanosoma cruzi* recombinant antigens. Rev. Soc. Bras. Med. Trop..

[152] Miyahira Y., Katae M., Kobayashi S., Takeuchi T., Fukuchi Y., Abe R., Okumura K., Yagita H., Aoki T. (2003). Critical contribution of CD28–CD80/CD86 costimulatory pathway to protection from *Trypanosoma cruzi* infection. Infect. Immun..

[153] Dutra W.O., Martins-Filho O.A., Cançado J.R., Pinto-Dias J.C., Brener Z., Gazzinelli G., Carvalho J.F., Colley D.G. (1996). Chagasic patients lack CD28 expression on many of their circulating T lymphocytes. Scand. J. Immunol..

[154] Menezes C.A., Rocha M.O., Souza P.E., Chaves A.C., Gollob K.J., Dutra W.O. (2004). Phenotypic and functional characteristics of CD28^+^ and CD28^−^ cells from chagasic patients: Distinct repertoire and cytokine expression. Clin. Exp. Immunol..

[155] Martins G.A., Campanelli A.P., Silva R.B., Tadokoro C.E., Russo M., Cunha F.Q., Rizzo L.V., Silva J.S. (2004). CD28 is required for T cell activation and IFN-gamma production by CD4^+^ and CD8^+^ T cells in response to *Trypanosoma cruzi* infection. Microbes Infect..

